# NOVAprep-miR-Cervix: New Method for Evaluation of Cervical Dysplasia Severity Based on Analysis of Six miRNAs

**DOI:** 10.3390/ijms24119114

**Published:** 2023-05-23

**Authors:** Margarita Kniazeva, Lidia Zabegina, Andrey Shalaev, Olga Smirnova, Olga Lavrinovich, Igor Berlev, Anastasia Malek

**Affiliations:** 1 Subcellular Technology Lab., N.N. Petrov National Medical Research Center of Oncology, 197758 St. Petersburg, Russia; margo9793@gmail.com (M.K.); lidusikza@yandex.ru (L.Z.); shalaeff.andrey@gmail.com (A.S.); 2 Department of Gynecological Oncology, N.N. Petrov National Medical Research Center of Oncology, 197758 St. Petersburg, Russia; ssmirnova.oa@gmail.com (O.S.); olgalav1973@mail.ru (O.L.); iberlev@gmail.com (I.B.)

**Keywords:** cervical cancer, intraepithelial neoplasia, diagnosis, miRNA, RT-PCR, random forest

## Abstract

Cervical cancer is one of the most common gynecological malignancies and it is preventable through the yearly diagnosis and management of pre-cancerous cervical disease. The profile of miRNA expression in cervical epithelium cells is altered with cervical dysplasia development and further progression. The NOVAprep-miR-CERVIX is a new approach for the assessment of cervical dysplasia through the analysis of six marker miRNAs. This study aims to evaluate theperformance and diagnostic potency of the new method. Cytological smears from 226 women (NILM, n.114; HSIL, n.112) were included in the study. A VPH test was performed with RealBest DNAHPV HR screen Kit, six marker miRNAs (miR-21, -29b, -145, -451a, -1246, -1290) were assayed using NOVAprep-miR-CERVIX kit. Obtained data were analyzed using the Delta Ct method and random forest machine learning algorithm. The results of the quantitative analysis of six microRNAs were expressed as a miR-CERVIX parameter, which ranged from 0 to 1, where “0” corresponded to the healthy cervical epithelium, while “1” corresponded to high-grade squamous intraepithelial dysplasia. The average value of miR-CERVIX differed in groups of NILM and HSIL samples (0.34 vs. 0.72; *p* < 0.000005). An estimation of miR-CERVIX allowed for the differentiation between healthy and pre-cancerous samples with sensitivity of 0.79 and specificity of 0.79, as well as to confirm HSIL with specificity of 0.98. Interestingly, the HSIL group included HPV(+) and HPV(−) samples, which were statistically significantly different in terms of miR-CERVIX value. Analysis of CC-associated miRNAs in material of cervical smear might serve as an additional method for the evaluation of cervical dysplasia severity.

## 1. Introduction

The cervical cancer (CC) incidence rate has decreased considerably from the mid-1970s to the mid-2000s due to implementation of screening programs based on the cytological evaluation of cervical smears or/and HPV testing. Over the last decades, the technological performance and diagnostic efficacy of both the Pap test and HPV testing have been improving constantly. For instance, liquid-based cytology reduced the amount of unsatisfactory smears and increased the rate of CIN1/CIN2 detection compared to conventional cytology [1]. Moreover, evaluation of cervical cytology images can be improved by deep learning models [2], although PCR-based detection of HPV is quite sensitive and robust methods, new CRISPR/Cas9-based [3] or infrared spectroscopy-based [4] technologies, have been developed to advance HPV testing. Despite the implementation of standard Pap-test/HPV-test-based screening programs, the mortality rate of CC in the Russian Federation has dropped from 8.2 (2010) to only 7.9 (2020) cases per year per 100,000 [5]. Such modest results can be explained by two factors: low screening rate and innacuarcy of testing method. The imput of each factor can be estimated using the following example. In the region where the coverage of the population with the screening program was 95% over 5 years (Tambov oblast), a 2.6-fold decrease in the mortality rates was achieved [6]. These results highlighted the need to scale up the screening rate, but the mortality still observed with near-complete population screening pointed to the inherent limitations of Pap-test/HPV-test-based approach. For instance, it was indicated in annual reports of cytological laboratories in Sankt-Petersburg that Pap-test/HPV-test-based screening reveals that 5–8% of women have moderate to poorely characterized dysplasia of the cervical epithelium (LSIL, ASC, ASC-US, ASC-H). This relatively small group can make a significant contribution to the overall CC mortality. The correct management of these patients requires the development and application of new diagnostic approaches.

In addition to viral infection assessment, the identification of endogenous genetic and/or epigenetic markers of cervical carcinogenesis can make a qualitative contribution to CC prevention. For instance, comprehensive genomic profiling revealed a list of potential genetic markers of CC [7,8]: FAT1, HLA-B, PIK3CA, MTOR, KMT2D, ZFHX3, FAT1, MLL3, MLL2, and FADD. Genome-wide methylation profiling studies of CC cells revealed typical alterations. Several kits testing the methylation status of certain genes have already been evaluated and released to the market: QIAsure Methylation test/Qianen GmbH (FAM19A4, hsa-mir124-2) [9], GynTest assay/Oncgnostics GmbH (ASTN1, DLX1, ITGA4, RXFP3, SOX17, ZNF671) [10], PAX1 DNA Detection Kit/iStat Biomedical Co., Ltd. (PAX1) [11]. In contrast to the methylation status, there is no consensus on the CC-associated gene expression alterations. However, the expression changes of certain messenger RNAs (mRNAs), circular RNAs (circRNAs) or microRNAs (miRNAs) might certainly have diagnostic potency. Metaplasia, or the replacement of the columnar epithelium with squamous metaplastic epithelium, which occurs in the transformation zone, is assumed to be an initial carcinogenic event. Metaplastic cells are sensitive to different factors, such as vaginal microbiota, estrogen influence, and viral infection, which synergistically induce epithelial cell proliferation, alteration of paracrine stromal–epithelial interactions, and may eventually promote neoplastic progression [12]. These alterations are associated with specific changes in the cellular miRNA profile [13]. For example, the HPV-16 contributes in the progression of CC by regulating the miR-142/PD-L1 exis [14]; the intercellular adhesive properties of cervical epithelial cells are altered by miR-95 via the suppression of vascular adhesion molecule 1 (VCAM1) [15]. Interestingly, the stepwise progression of precancerous cervical epithelium changes is associated with an increase in the qualitative and quantitative alterations of miRNAs expression: increase in number of over/down-regulated miRNAs [16], as well as increase in the level of expression shift of certain miRNAs [17]. This phenomenon provides the basis for the development of a method for the accurate diagnosis and monitoring of the precancerous condition of the cervical epithelium. However, the development of miRNA-based diagnostic assays is hampered by a number of technical problems. The isolation and PCR-based quantification of miRNAs are not trivial issues because of the small size of these molecules, whereas the interpretation of PCR results is complicated by the absence of reliable normalization approaches and the presence of non-mature forms of marker miRNAs.

In our previous studies, we identified CC-associated miRNAs [17,18,19]. These results were combined with original published study analyses to select the potencial miRNA markers of cervical carcinogenesis (Table 1). We also explored the highly sensitive two-tailed RT-qPCR technology for miRNA quantification and evaluated a new approach for RT-PCR results normalization [20,21].

All these developments were used to create a new method for the cervical dysplasia evaluation based on an analysis of six CC-associated miRNAs. Additionally, a “random forest” machine learning algorithm was applied to improve the interpretation of the results. In the present work, we investigated the performance of the NOVAprep-miR-CERVIX kit and discussed its possible application for CC diagnosis. 

## 2. Results

### 2.1. Design of Study

**Figure 1:**
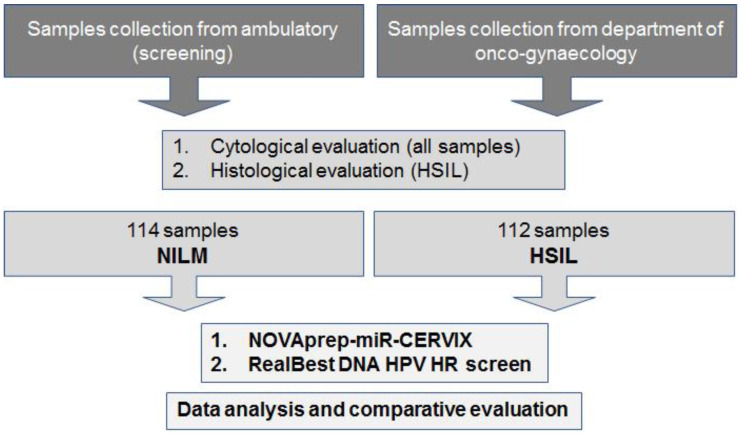
Study design.

The study aimed to evaluate the performance and diagnostic potency of the NOVAprep-miR-CERVIX kit, and to explore its possible application in clinical practice. We collected samples of normal cervical epithelium, NILM (n.114), and samples of histologically confirmed obligate precancerous epithelium, HSIL (n.112). The design is presented in Figure 1.

### 2.2. NOVAprep-miR-CERVIX Performance

The NOVAprep-miR-CERVIX kit was designed to perform quantitative analysis on eight molecules: six marker miRNAs and two controls. Potentially marker miRNAs were selected on the basis of the results of earlier published investigations and analysis of the scientific literature. Thus, the over-regulations of miR-145 and miR-1246 are associated with cervical dysplasia development, as reported previously [18,19]. The miR-1290 is a molecule involved in the development of squamous epithelial malignancies of various localizations, including oral and laryngeal squamous cell carcinoma [39]. We confirmed its over-expression in cervical cancer in our pilot study (data not published). We have previously reported the same level of miR-21 expression for both NILM and HSIL samples of air-dried cervical Pap smears [18], so this molecule was included in the NOVAprep-miR-CERVIX assay as a normalizer. In the pilot study, we observed a down-regulation of miR-451 and miR-29b in HSIL samples versus NILM (data not published), and these observations were confirmed by published results. The expression of miR-29b is reduced during cervical dysplasia development [27], whereas the re-activation of miR-29b activity might have a therapeutic effect [40,41]. The miR-451 behaved as a tumor suppressor in cervical cancer cells HELA [42]. Thus, the three miRNAs that were assumed to be over-regulated and the three miRNAs that were assumed to be stable or down-regulated during cervical carcinogenesis were included in the NOVAprep-miR-CERVIX.

Nucleic acids were isolated, as described in Section 2.2.; the sufficiency of the quality and quantity of nucleic acid was assayed by PCR analysis of the conserved non-translated region of the ACTB gene; samples with Ct_(ACTB_) > 34 were excluded from the analysis. In order to control the efficacy of enzymatic reactions, synthetic cel-miR-39-5p was mixed with each sample at a final concentration of 0,1 nM, and RT-qPCR analysis of this molecule was included in the test. The expected value of Ct_(cel-miR39)_ was under 18.5, and the samples with higher values were considered as non-appropriated. Thus, a complete analysis of one sample included eight reactions; an assay for twelve samples included two 96-well plates (RT and PCR) designed and manufactured by Algimed Techno Ltd. (Minsk, Belarus). The 226 samples were enrolled in the analysis; however, the appropriate results of both control reactions were obtained from 188 samples. Thus, the results of the NOVAprep-miR-CERVIX analysis were eligible for further evaluation and interpretation in 83% cases only. HPV testing was performed as an independent assay and was resultative in all cases. Thus, HPV testing turned out to be a more robust technology than miRNA analysis.

### 2.3. NOVAprep-miR-CERVIX Results Evaluation

For each sample that passed the quality control, Ct values for six miRNAs were obtained (Supplementary Table S1). The next step of analysis was a calculation of amplification ratios for reciprocally regulated miRNAs pairs using the standard approach:

$$dCt=\left(2^{Ct\left(miRX\right)-Ct\left(mirY\right)}\right)$$

**Figure 2:**
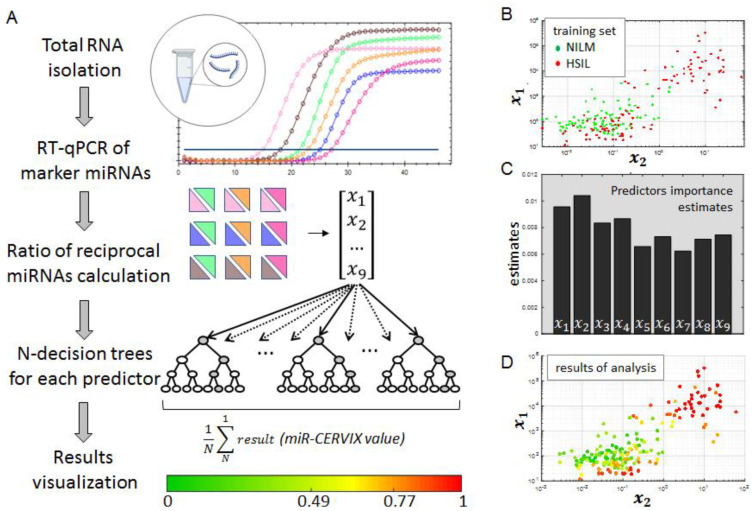
Analytic algorithm of NOVApre-miR-CERVIX kit and example of its evaluation. (**A**) Schematic workflow of analysis includes six RT-PCR reactions, estimation of nine predictors (χ1 − χ9), application of machine learning algorithm to identify input of each predictor, and calculation of miR-CERVIX value. (**B**) Visualization of input data (clinical diagnosis) distribution in accordance with the two predictors, χ1 and χ2 values, as examples; each point reflects individual sample with morphological diagnosis NILM (green) and HSIL (red). (**C**) Histogram reflecting input of each one of the nine (χ1–χ9) predictors to estimate the miR-CERVIX value. (**D**) Visualization of output data (predicted diagnosis) distribution based on the two predictors; each point reflects individual sample colored in accordance with miR-CERVIX value.

We have shown the effectiveness of this approach in several previous studies [20,21]. Each of the three miRNAs that were expected to be up-regulated in HSIL (miR-145, miR-1246 and miR-1260) were normalized versus each of the three normalizers (miR-21, miR-29b, miR-451). Thus, nine rations, herein referred to as predictors, (x_1–9_) were calculated for each samples. The workflow of analysis is schematically shown in Figure 2A.

Next, we developed an approach for ranking the nine predictor values and interpreting the results. To create a diagnostic model, we used machine learning methods, in particular, the random forest (RF) algorithm. The RF algorithm is suitable for solving a wide range of problems: classification, regression, clustering, feature selection, etc. The algorithm allows us to work with a large number of features and classes, to evaluate the contribution of individual features to the model and to process data with missing feature values [43]. The model used a collection of individual learners, decision trees for our case, for making predictions on new data. The algorithm was based upon bootstrap aggregation combined with ensemble learning. Tree ensemble were generated using the fitensemble function by following input arguments; nine values, χ1–χ9, were used as predictors, and verified diagnoses were used as responses. For a better *understanding* of the results, we demonstrated a graph where the axes corresponded to χ1 and χ2 values. Figure 2B shows data for the training set: red dots show samples with HSIL, green dots show NILM samples. Before the main analysis, we evaluated the contribution of each predictor to the formation of the final value (Figure 2C). To improve the accuracy of the model and to avoid overfitting, the 20 folded (kfold = 20) cross-validation (CrossVal) function was included in the model. This function randomly partitioned data into k sets, trained the model on the k − 1 folds of the training data, and validated the resulting model on the rest of the data. The resulting graph was built using the same coordinates as the training dataset. Subsequently, all the results were averaged to create a single estimation. The result of the modelling was a number in the range from 0 to 1 (herein referred to as the miR-CERVIX value), where “0” reflected a confidently normal condition, whereas “1” reflected a high-grade intraepithelial squamous lesion (Figure 2D). We have provided a color scale to simplify the clinical interpretation of the results and make it more intuitive.

### 2.4. Correlation of miR-CERVIX Value and Morphological Data

**Figure 3:**
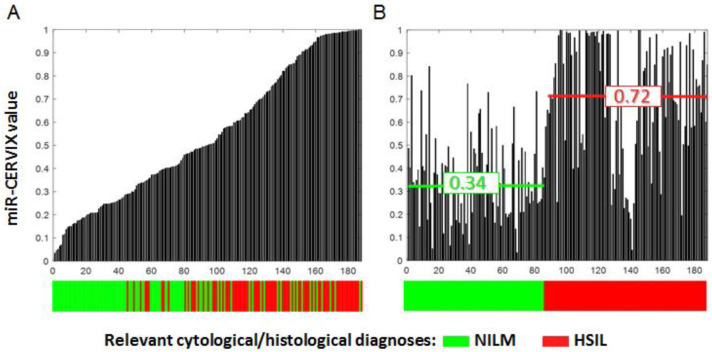
Combined visualization of miR-CERVIX value and morphological examination results. Black bars in histogram reflect miR-CERVIX value (varied from 0 to 1), whereas color line below reflects morphological diagnosis (either NILM or HSIL). (**A**) The samples (n.188) are arranged in ascending order of miR-CERVIX value. (**B**) The samples are distributed in accordance with morphological diagnosis. Numbers reflect averaged miR-CERVIX values in groups of NILM and HSIL samples. The difference was statistically significant (*p* < 0.000005), as estimated by Mann–Withney test.

To access the quality of the developed model, we compared the obtained results and the results of the morphological evaluation of cervical smears. When the results were ranked in the ascending order of the miR-CERVIX value, expressed by black bars on the graph, the corresponding cytological/histological diagnoses were indicated on the colored bar below the graph (Figure 3A).

Figure 3A clearly indicates the correlation between the two parameters; however, there were both NILM samples (green bars) with a high value of miR-CERVIX, and HSIL samples (red bars) with a low value of miR-CERVIX. The distribution of the results with an alternative principle, where samples are ordered according to cytological/histological diagnoses, is shown in Figure 3B. The average miR-CERVIX value for group of NILM samples (n.87) was 0.34, and the average miR-CERVIX value for a group of HSIL samples (n.101) was 0.72. The difference between the groups, as estimated with the Mann–Withney test was statistically significant (*p* < 0.000005). This result revealed the difference in the miRNA profiles between the comparison groups; however, the large number of discrepancies between the morphological diagnoses and the miR-CERVIX values still indicated the imperfection of both the diagnostic approach itself and the method of its validation. The subjective character of the cytological/morphological diagnosis, as well as the natural variability within a groups of equally categorized samples should be considered as a possible factor of diagnostic discrepany. Moreover, the miRNAs expression profile might closely reflect the complex and stepwise process of the cervical epithelium transformation. If this is the case, then a pattern of six marker miRNAs converted into an miR-CERVIX value may better match the biological status of epithelial cells than the four-level Bethesda system. More investigations are required to evaluate the biological and clinical significance of the observed discrepancy between the morphological diagnosis and miR-CERVIX value. 

### 2.5. Possible Application of NOVAprep-miR-CERVIX

**Figure 4:**
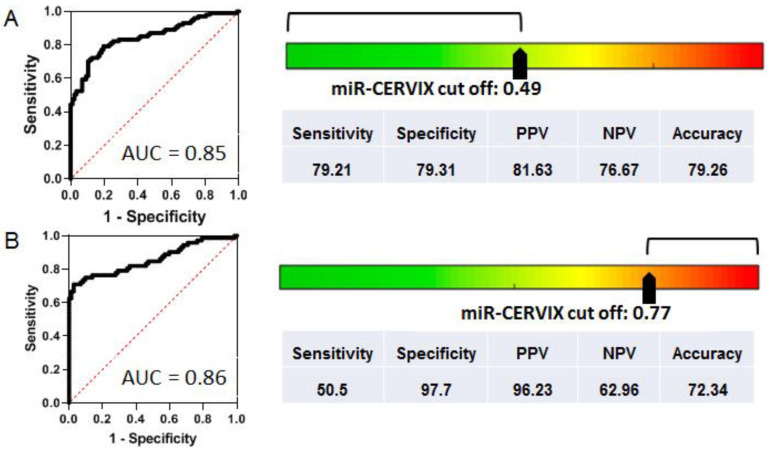
Examples of possible application of NOVAprep-miR-CERVIX. (**A**) The method can be used to distinguish between normal and pathological specimens by setting a miR-CERVIX cut-off value of 0.49: ROC curve, AUC value, and parameters of diagnostic potency were estimated using cytological/gistological diagnosis as reference. (**B**) The method can be used to confirm cases of HSIL by setting a miR-CERVIX cut-off value of 0.77: ROC curve, AUC value and parameters of diagnostic potency were estimated in the same way.

It is still difficult to define the optimal place for NOVAprep-miR-CERVIX in CC screening/diagnostic algorithms. However, we hope that the developed method can be used to solve certain clinical problems. For instance, it can be helpful for the objective evaluation of samples from a cohort of young women, when the transient HPV infection is common and the diagnostic value of HPV testing is low. A diagnostic algorithm can be tuned to distinguish between normal and pathological status by defining a miR-CERVIX threshold of 0.49. To define the diagnostic potential of NOVAprep-miR-CERVIX, we used the morphological evaluation of cervical smears as a method of reference, and standard parameters (sensitivity, specificity, positive predictive value, negative predictive value, accuracy, and precision) were estimated. The result are presented in Figure 4A. The pathological (precancerous) condition can be distinguished from normal status with sensitivity of 0.79 and specificity of 0.8, AUC = 0.85.

Another clinical problem is achieving a confident and accurate selection of patients with HSIL for cervical surgery. The miR-CERVIX threshold shifted to 0.77, and would allow the confirmation of the diagnosis of HSIL with very high specificity (0.97), as shown in Figure 4B. This additional diagnostic technology can be very helpful when cervical surgery is being considered as method of HSIL therapy for young patients with reproductive plans.

Thus, NOVAprep-miR-CERVIX appeared to provide flexible tool for the assessment of the cervical epithelium status; however, its optimal application should be defined in an additional study.

### 2.6. Comparison NOVAprep-miR-CERVIX and HPV Testing

**Figure 5:**
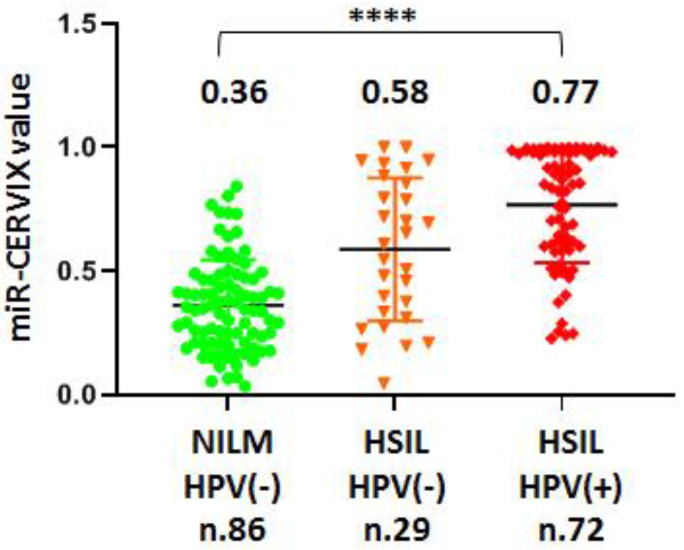
Comparative evaluation of miR-CERVIX value. Samples are grouped on the basis of morphological diagnosis and HPV testing: NILM/HPV(−), HSIL/HPV(−), and HSIL/HPV(+). The miR-CERVIX value is reflected along the y-axis. The miR-CERVIX values averaged within each group are indicated in the graph. The difference between the three was statistically significant, as estimated by the Kruskal–Wallis test and indicated as **** (*p* < 0.00005).

All samples included in the study were tested for HPV infection. We used a screening test (RealBest DNA HPV HR screen) that detected fourteen carcinogenic serotypes of HPV (16, 18, 31, 33, 35, 39, 45, 51, 52, 56, 58, 59, 66, and 68) without differentiation. Group NILM samples were almost completely negative for HPV (one positive samples from 87), while the HSIL group included 72 HPV(+) samples and 29 HPV(−) samples. These were unexpected results, contradicting the generally accepted opinion that the human papilloma virus is always involved in cervical carcinogenesis. Moreover, the average miR-CERVIX value in the group of HPV(−) samples of HSIL (n.29) was significantly lower compared to the group of HPV(+) samples with the same cito-/histological status, 0.58 vs. 0.77 (Figure 5).

There is significant overlap between miR-CERVIX values in comparison groups; therefore, the miR-CERVIX value cannot be considered as diagnostic criterion here. However, the miR-CERVIX value seemed to reflect the stepwise process of the miRNAs’ expression shift from NILM to HSIL/HPV(−), and further toward HSIL/HPV(+). Moreover, the obtained results may indicate a difference in the status of HPV(−) and HPV(+) samples of HSIL, which confirmed that the identification of HSIL/HPV(−) group due to analytic errors in HPV testing was not random.

In order to directly compare the diagnostic potency of the two methods (NOVAprep-miR-CERVIX and HPV testing), the standard parameters were estimated using morphological diagnosis as a reference (Table 2).

For example, when the parameter of miR-CERVIX was set to 0.49, NOVAprep-miR-CERVIX defined the precancerous alterations of cervical epithelium with higher sensitivity (79.21 vs. 71.29), whereas they were defined with lower specificity (79.31 vs. 98.85) compared to HPV testing.

## 3. Discussion

### 3.1. General Aspects of miRNA-Based Test

Accumulating evidence demonstrates that miRNAs play an important role in cervical carcinogenesis and might serve as new promising markers of pre-cancerous alterations of the cervical epithelium. In order to develop an effective miRNA-based diagnostic approach, we attempted to solve two main tasks: (1) To create a robust analytic technology; and (2) To evaluate its performance in comparison with existing diagnostic methods. The developed method has two principal innovations. First, it is objective in contrast to cytological or histological evaluations that are depend on the personal experience of pathologists. Second, it reflects a proper biological status of the cervical epithelial cells in contrast to HPV testing, which indicates just a presence of infection, which is clearly transient. Results reported herein revealed certain achievements; however, we discovered several challenges and indicated some approaches for optimization.

### 3.2. The Main Approaches for the Improvement of NOVAprep-miR-CERVIX and Plans for the Future

#### 3.2.1. Optimal Selection of Marker miRNAs Using NGS

Robust diagnostic technology requires the right selection of diagnostic markers, a sensitive and specific method for its quantification, and an effective approach for data interpretation. In our study, the selection of six marker miRNAs was based on the preliminary investigations and the results achieved by other groups. However, the results of CC-associated miRNA profiling published so far are very heterogeneous; therefore, our selection of potential miRNA markers was not very confident. The variability of available miRNA profiling data can be explained by the variability of the used platform: Agilent’s miRNA microarray [44,45], The TaqMan^®^ Array Human MicroRNA [46,47], Affymetrix miRNA array [48], NanoString Technologies [49], and HiSeq 2500 Sequencing System [50,51]. An additional factor is the biological plasticity of cervical squamous epithelium; the miRNA profile of epithelial cells is mediated by the maturity stage and cyclic hormonal control. A huge number of biological samples is required to identify these cells’ common characteristics, which is difficult to perform in a single study. We believe that the suboptimal choice of miRNA markers is the main disadvantage of the NOVAprep-miR-CERVIX system and the main opportunity for improvement. In the future, we plan to perform NGS-based miRNA profiling on a sufficient number of samples, and to select the reciprocally regulated miRNA pairs (or predictors χ) with the highest diagnostic value using the full sequencing dataset.

#### 3.2.2. Optimization of Analytic Algorithm by Increasing Training Set

The quantification of six selected miRNAs was performed by two-tailed RT-qPCR [52]. This technology has worked well in our previous studies, but the lack of methods for the interpretation of PCR results has always hindered the clinical implication of our products. We believe that the development of an innovative algorithm for the RT-qPCR result interpretation is most important achievement of our study. The absence of a robust method for normalizing of miRNA RT-qPCR data is well-known problem that still has no solution. Due to the tissue-specific pattern of miRNA expression [53], there are options either to use small nuclear RNA or to identify stably expressed miRNA in tissue of interest. Both approaches are doubtful: the snoRNAs expression can be associated with tumor pathology [54], the “house-keeping” miRNAs for cervical normal cervical epithelium have not yet been identified, whereas miRNAs proposed as optimal references for CC (miR-151-5p, miR-152-3p, and miR-423-30) [50] were not confirmed through our research. Thus, the identification of reciprocally dysregulated miRNAs appeared to be the method of choice, which we applied previously [20,21]. To increase the diagnostic potency of this approach, we explored three up-regulated and three stable/down-regulated miRNAs, defined nine predictors (χ), and developed a computational algorithm (random forest) for their complex evaluation. As far as we know, this was the first attempt at such a solution, and it was justified by the obtained results. Since the quality of a machine learning algorithm depends on the size of the training sample, a larger multicenter study is needed to optimize the existing analytic approach.

#### 3.2.3. Comparison with Other Molecular Diagnostic Approaches

To evaluate the performance of NOVAprep-miR-CERVIX, we compared it with traditional methods for a cervical dysplasia diagnosis: cervical cytology and HPV testing. Given the objective limitations of the cervical cytology, only well-distinguished conditions were included in our study, each specimen was independently evaluated by two qualified cytologists, and all HSIL cases were histologically confirmed. Even with such strong selection, neither NILM (n.87) nor HSIL (n.101) were homogeneous in terms of miR-CERVIX value (Figure 3A,B). These results indicated the non-exhaustive character of morphological classification and the heterogeneity of samples within morphologically identical groups. Thus, a comprehensive evaluation of the clinical utility of NOVAprep-miR-CERVIX will require its direct comparison with relevant commertially available tests, such as CINtec/Roche Diagnostic, GynTest/Oncgnostics, QIAsure Methylation Test/Qiagen or PAX1 DNA Detection Kit/iStat Biomedical Co. Ltd.

#### 3.2.4. Search for Appropriate Clinical Application and Relevant Design of Clinical Study

Within this study, the standard parameters of the diagnostic potency of NOVAprep-miR-CERVIX were estimated using cytological/histological diagnosis as reference. Since the result of the proposed test has a quantitative character (miR-CERVIX value varied from 0 to 1), we estimated these parameters with different cut-off rates. For instance, assuming the purpose of the investigation is to distinguishing healthy status individuals from individuals with dysplasia, the cut-off value of miR-CERVIX was set at 0.49. In this case, the HSIL will be diagnosed with sensitivity of—0.79 and specificity of—0.97. In contrast, assuming the purpose of the investigation was the confident indication of cervical surgery when cytology is doubtful and the patient is HPV(+), the cut-off value of miR-CERVIX was set to 0.77. In the assumed situation, the evaluation of the miR-CERVIX value may be helpful in avoiding an aggressive curative approach in cases of young patients with reproductive plans. To determine the appropriate clinical application of the NOVAprep-miR-CERVIX kit, we plan to study its prognostic value in the cases of intermediate cytological status (LSIL, ASC, ASC-US, and ASC-H). This will require a dynamic miR-CERVIX value estimation in parallel with cytology and HPV testing over a period of 12-24-36 months. Additionally, cases with discordant results of cytology and HPV testing present an interesting object for further research.

### 3.3. Possible Causes and Interpretaion of High Rate of HPV-Negative HSIL Samples

As we reported, 29% of those in the HSIL group (n.101) tested negative for HPV. This was an unexpected result, contradicting the generally accepted trigger role of HPV in cervical carcinogenesis. It has been stated that HSIL is caused by HPV infection and the viral mechanisms of carcinogenesis are well studied and overall accepted [55]. However, its presence does not always mean that it is involved; similarly, the understanding of some mechanisms does not exclude the existence of others. Two arguments supported our confidence in the obtained results. First, the statistically significant difference of the miR-CERVIX value between HSIL-HPV(+) and HSIL-HPV(−) groups indicated a real biological difference between them, not just random distribution due to analytical errors caused by the HPV test. Second, there is no explanation as to why HPV infection plays a crutial role in CC, and why it is so rarely detected in other types of squamous cell carcinoma. The similar process of squamous epithelium carcinogenesis in the oral cavity is associated with HPV in 4% of cases [56], and in the head and neck zone it is associated with 20% of cases [56] only. Our investigation was not aimed at reconsidering the role of HPV in CC, and it did not provide results that sufficiently indicate this. However, looking at Figure 5, we can suppose that both the viral infection and the marker miRNAs expression shifts are being aggravated by cervical dysplasia progression. Complex assessment of both parameters might provide useful diagnostic tools. However, this assumption will require further research with an extensive evaluation of the HPV infection status, which is out of the scope of our research.

### 3.4. Concluding Remarks

Overall, our results provided new pieces of evidence that the four-level Bethesda classification (NILM, ASCUS, LSIL, HSIL) may represent a spectrum where overlaps invariably exist, which reduce the efficacy of standard Pap testing and CC screening. In our opinion, there are two main approached in modifying the CC screening strategy. First, to make the standard Pap-test/HPV-test-based screening cheaper and more widespread, possibly by reducing its specificity. Second, to make different methods of in-depth analysis available to the high-risk groups identified during the course of screening. The spectra of these method should include the analysis of proteins associated with abnormal proliferations (e.g., CINtec), methylation status (e.g., GynTest or PAX1 DNA methylation kit), the profiling of marker miRNA and so on. The best application for each of these tests will be defined in practice.

## 4. Materials and Methods

### 4.1. Patients and Samples

The study was approved by the Local Ethic Committee of the Petrov’s NMRC of oncology (protocol №27/27, 18 January 2021). An informed consent form was signed by each patient. Inclusion criteria are an age ranging from 25 to 50 years old and having the ability to undergo all the required tests (gynecological investigation, liquid cytology, HPV test, and NOVAprep-miR-CERVIX test). Pregnant and menopausal women, patients with metabolic and autoimmune diseases, or who had any malignancies in the past, as well as patients who had undergone any cervical surgery were excluded from the study. The cervical scrapings were prepared by routine Papanicolaou staining. The cytological examination of all samples was performed independently by two qualified cytologists. The samples were classified according to the Bethesda system. To reduce the number of errors in cytological diagnostics, cases were excluded from further analysis when at least one of the two cytologists observed signs of precancerous abnormalities in the cervical epithelium, such as a low-grade squamous intraepithelial lesion (LSIL), atypical squamous cells (ASCs), atypical squamous cells with undetermined significance (ASC-US) or atypical squamous cells, HSIL cannot be excluded (ASC-H). We included in the study only those cases where both cytologists confidently confirmed the absence of neoplastic findings (negative for intraepithelial lesions or malignancy, NILM) or a diagnosed high-grade squamous intraepithelial lesion (HSIL). All patients with HSIL were referred to colposcopy; biopsy of abnormal zones was performed to confirm the diagnosis of HSIL.

### 4.2. Nucleic Acids (NAs) Isolation

After standard staining and cytological evaluation, the suspension of exfoliated cervical epithelial cells (1.5 mL) resting in the liquid cytology vial were transferred into 2 mL tubes, cells were sedimanted from suspension by centrifugation, and a pellet was used for total RNA extraction in accordance with a standard magnetic bead-based protocol, including the binding of NA with magnetic beads, washing, and elution according to the manufacturer’s protocol. Samples with RNA concentration above 100 ng/µL and absorption rate of 260/280 above 1.7 were included in the study. The concentration of DNA was not measured while it was estimated using a copy number assessment of a conserved non-translated region of the β-actin gene (ACTB) by real-time PCR.

### 4.3. MiRNA Analysis and HPV Testing

NOVAprep-miR-CERVIX (Algimed Techno Ltd. Minsk, Belarus) kit was applied to quantify six marker miRNAs (miR-21-5p, miR-29b-3p, miR-145-5p, miR-451a-5p, miR-1246-5p, and miR-1290-3p) by a two-tailed reverse transcription, followed by quantitative PCR (ttRT-qPCR). In parallel, the reaction of amplification of the unique genomic region (material quality/quantity control) and the RT-qPCR analysis of a synthetic version of C.elegans cel-miR-39-3p (spike-in control) were performed for each sample. The sequences of primers for ttRT-qPCR are presented in Supplementary Table S2. The HPV testing was performed with the RealBest DNAHPV HR screen Kit (Vector Best, Novosibirsk, RF) for the detection of human papillomavirus types 16, 18, 31, 33, 35, 39, 45, 51, 52, 56, 58, 59, 66, 68 DNA without differentiation. The CFX96 Touch™ Real-Time PCR Detection System (Bio-Rad Laboratories Inc, Hercules, CA, USA) was used for all reactions. The HPV testing results were interpreted in accordance with the manufacturer’s manual and expressed as either (+) positive or (−) negative.

### 4.4. RT-PCR Data Analysis and Interpretation

To evaluate the result of the NOVAprep-miR-CERVIX test, we used following algorithm. First, samples with non-appropriate results of control reactions were excluded from further analysis. Next, amplification ratios of reciprocally dysregulated miRNAs pairs (n.9) were calculated using the standard approach for each sample. Finally, we used the random forest machine learning algorithm, implemented in the MatLab R2016a (The MathWorks, Natick, USA), to develop method of nine values for complex evaluation. We used the ‘fitensemble’ function, which allowed us to create an ensemble of decision trees for a randomly selected subset from the training data for several randomly selected predictors from the total set. Thus, the set of 188 values was used for 9 predictors to train the model. The result of the algorithm was to obtain a value from 0 to 1 for each element of the test set.

The Mann–Withney test was used to evaluate the statistical significance of the miR-CERVIX difference between clinical groups (NILM vs. HSIL). The standard indicators (sensitivity, specificity, positive predictive value, negative predictive value, and accuracy) were used to estimate the diagnostic potency of the developed method.

## Figures and Tables

**Table 1:** MiRNAs selected as potential markers of CC for testing by NOVAprep-miR-CERVIX kit.

miRNA/ID	References
hsa-miR-21-5p (MIMAT0000076)	[22,23,24]
hsa-miR-29b-3p (MIMAT0000100)	[25,26,27]
hsa-miR-145-5p (MIMAT0000437)	[28,29,30]
hsa-miR-451a-5p (MIMAT0001631)	[31,32,33]
hsa-miR-1246-5p (MIMAT0005898)	[34,35,36]
hsa-miR-1290-3p (MIMAT0005880)	[37,38]

**Table 2:** Parameters of diagnostic potency of miR-CERVIX and HPV testing.

	Sensitivity	Specificity	PPV	NPV	TP	FP	TN	FN	Total
miR-CERVIX (0.49)	79.21	79.31	81.63	76.67	80	18	69	21	188
miR-CERVIX (0.77)	50.5	97.7	96.23	62.96	51	2	85	50	188
HPV screen	71.29	98.85	98.63	74.78	72	1	86	29	188

## Data Availability

Data is contained within the article or supplementary material.

## Supplementary Materials

The following supporting information can be downloaded at: https://www.mdpi.com/article/10.3390/ijms24119114/s1.
