# Supplementary material for: NOVAprep-miR-Cervix: New Method for Evaluation of Cervical Dysplasia Severity Based on Analysis of Six miRNAs
Source: Int J Mol Sci. 2023 May 23;24(11):9114. doi: 10.3390/ijms24119114 (PMC10252283; doi:10.3390/ijms24119114)
Supplement: Supplementary file 1 [file ijms-24-09114-s001.zip › Suppl. Table S1.pdf]

NOVAprep-miR-Cervix: New Method for Evaluation of Cervical Dysplasia Severity Based on Analysis of Six miRNAs

Margarita Kniazeva, Lidia Zabegina, Andrey Shalaev, Olga Smirnova, Olga Lavrinovich, Igor Berlev and Anastasia Malek

Table S1. Sequences of oligonucleotides used within NOVAprep-miR-CERVIX assay.

miRNA	Primers	Sequence of primers 5′→3′
miR-21	RT-primer	ATAAGCTACAACGACCAGAGCTAGAGAACCTAGCTCACCCACTACTCAACA
	forward primer	CATAAGCTACAACGACCAGAG
	reverse primer	GGTAGCTTATCAGACTGATGT
	probe	FAM-AGAGAACCTAGCTCACCCACTAC-BHQ1
miR-29b	RT-primer	CAAATGGTCGACGAATACTGCTAGAGTTGCTAGCAGAGCCCTTAAAACACT
	forward primer	CAAATGGTCGACGAATACTG
	reverse primer	GGTAGCACCATTTGAAATCAG
	probe	FAM-AGAGTTGCTAGCAGAGCCCTTAA-BHQ1
miR-145	RT-primer	AACTGGACCGACGAATACTGCTAGAGTTGCTAGCAGAGCCCTTAAGATTCC
	forward primer	AACTGGACCGACGAATAC
	reverse primer	TCGTCCAGTTTTCCCAG
	probe	FAM-AGAGTTGCTAGCAGAGCCCTTAA-BHQ1
miR-451a	RT-primer	AACGGTTTCGACGAATACTGCTAGAGTTGCTAGCAGAGCCCTTAAAACTCA
	forward primer	CAACGGTTTCGACGAATAC
	reverse primer	GGAAACCGTTACCATTACTG
	probe	FAM-AGAGTTGCTAGCAGAGCCCTTAA-BHQ1
miR-1246	RT-primer	AATCCATTCAACGACCAGAGCTAGAGAACCTAGCTCACCCACTACCCTGCT

	forward primer	AATCCATTCAACGACCAGA
	reverse primer	GCACGAATGGATTTTTGGA
	probe	FAM-AGAGAACCTAGCTCACCCACTAC-BHQ1
miR-1290	RT-primer	AAAATCCACTATGCTCTCCAGGTACAGTTGGTACCTGTCTCCACTTTCCCTG
	forward primer	AAAATCCACTATGCTCTCCA
	reverse primer	CCGTGGATTTTTGGATCAG
	probe	FAM-TACAGTTGGTACCTGTCTCCACTT-BHQ1
cel-miR-39	RT-primer	TACACCCGCTATGCTCTCCAGGTACAGTTGGTACCTGTCTCCACTTCAAGCT
	forward primer	TACACCCGCTATGCTC
	reverse primer	GGCGGGTGTAAATCAG
	probe	FAM-TACAGTTGGTACCTGTCTCCACTT-BHQ1
b-actin	forward primer	CTGTTTTGTGGCTTGTTCAG
	reverse primer	AGGAAACCTTCCCTCCTCTA
