# Supplementary material for: NOVAprep-miR-Cervix: New Method for Evaluation of Cervical Dysplasia Severity Based on Analysis of Six miRNAs
Source: Int J Mol Sci. 2023 May 23;24(11):9114. doi: 10.3390/ijms24119114 (PMC10252283; doi:10.3390/ijms24119114)
Supplement: Supplementary file 1 [file ijms-24-09114-s001.zip › Suppl. Table S2.pdf]

NOVAprep-miR-Cervix: New Method for Evaluation of Cervical Dysplasia Severity Based on Analysis of Six miRNAs

Margarita Kniazeva, Lidia Zabegina, Andrey Shalaev, Olga Smirnova, Olga Lavrinovich, Igor Berlev and Anastasia Malek

Table S2. Complete results of NOVAprep-miR-CERVIX assay.

Samples that did not pass the control and excluded from the analysis are highlighted in gray

Sample №	Morphological diagnosis	NOVAprep-miR-CERVIX / results of individual marker quantification (Ct)								VPH test	miR-CERVIX
		b-actin	cel-miR-39-3p	hsa-miR-21-5p	hsa-miR-29-3p	hsa-miR-145-5p	hsa-miR-451a-5p	hsa-miR-1246-5p	hsa-miR-1290-3p		
1	NILM	30,40	16,48	25,05	31,02	29,53	26,28	19,57	27,73	NEGAT	0,49
2	NILM	33,02	16,27	23,45	34,23	28,62	32,46	15,54	25,66	NEGAT	0,40
3	NILM	32,52	18,11	30,73	34,51	29,79	34,24	16,88	23,87	NEGAT	0,80
4	NILM	31,92	16,12	26,13	27,52	30,18	22,14	19,32	26,15	NEGAT	0,34
5	NILM	32,18	16,19	25,99	27,77	31,64	21,12	20,19	27,95	NEGAT	0,33
6	NILM	31,73	15,87	24,45	31,42	27,90	32,27	18,36	26,42	NEGAT	0,35
7	NILM	33,13	17,17	24,59	34,58	29,91	32,40	15,31	24,01	NEGAT	0,39
8	NILM	32,20	16,60	24,21	25,52	28,84	22,19	16,88	23,77	NEGAT	0,15
9	NILM	30,51	17,78	30,30	31,14	29,57	32,92	19,15	24,81	NEGAT	0,74
10	NILM	32,06	17,02	24,58	31,31	32,09	33,35	17,31	24,48	NEGAT	0,41

11	NILM							29,44	23,11	NEGAT	
12	NILM		20,17	30,82		37,83	37,58	18,73	23,47	NEGAT	
13	NILM	39,57	17,68	25,48	24,82	29,63	27,85	14,47	20,43	NEGAT	
14	NILM	27,30	16,16	24,78		29,46	33,90	15,09	24,73	NEGAT	0,39
15	NILM		20,92	32,17	31,92	34,50	33,84	14,47	20,25	NEGAT	
16	NILM	32,36	18,07	27,17	29,61	32,06	31,38	16,28	22,19	NEGAT	0,55
17	NILM	28,17	15,80	23,31	29,80	24,92	26,03	16,14	25,17	NEGAT	0,18
18	NILM	28,36	17,20	27,73	29,65	27,04	31,33	17,18	24,06	NEGAT	0,84
19	NILM	30,81	17,42	25,59	25,79	30,33	23,17	19,12	26,26	NEGAT	0,25
20	NILM		42,91					22,05	25,52	NEGAT	
21	NILM	31,21	16,46	23,38	24,24	27,58	22,46	15,36	24,16	NEGAT	0,05
22	NILM	33,07	16,77	22,78	33,40	30,02	31,09	16,37	25,38	NEGAT	0,38
23	NILM		21,25	40,56	32,17	38,48	22,07	18,89	23,81	NEGAT	
24	NILM		33,61	28,06			26,18	20,14	27,26	NEGAT	
25	NILM	32,45	16,61	23,69	26,22	30,63	25,77	18,55	26,05	NEGAT	0,53
26	NILM		20,04	31,35	35,44	34,55	38,03	19,41	24,31	NEGAT	
27	NILM		19,36	32,67	31,70		32,98	18,00	23,51	NEGAT	
28	NILM	28,64	16,10	24,07	28,20	24,15	25,31	16,98	25,74	NEGAT	0,37

29	NILM	31,39	16,34	23,20	27,93	29,94	30,10	17,06	25,85	NEGAT	0,29
30	NILM	29,15	18,84	30,44	31,27	29,76	32,72	17,80	24,99	NEGAT	
31	NILM	32,21	17,46	22,13	29,37	30,27	26,15	15,15	22,87	NEGAT	0,29
32	NILM	32,68	16,49	23,13	24,46	30,13	20,58	17,23	26,02	NEGAT	0,42
33	NILM	30,20	16,25	25,37	26,03	31,08	24,27	19,27	26,16	NEGAT	0,12
34	NILM	30,34	17,84	22,29	32,27	29,28	32,75	16,47	24,70	NEGAT	0,41
35	NILM	27,85	16,30	24,12	32,30	28,14	32,37	14,91	27,04	NEGAT	0,41
36	NILM	28,78	16,47	21,51	23,02	25,34	21,17	15,33	22,91	NEGAT	0,49
37	NILM	31,55	18,74	24,30	30,85	28,94	31,59	15,78	24,50	NEGAT	
38	NILM	33,42	17,10	25,37	26,52	30,91	29,30	18,94	25,75	NEGAT	0,07
39	NILM		20,91	30,60	32,81	30,93	29,94	16,30	19,79	NEGAT	
40	NILM	29,87	16,79	23,49	24,57	29,67	20,39	16,31	23,00	NEGAT	0,15
41	NILM		38,58	34,72		35,88	33,76	17,41	22,86	NEGAT	
42	NILM	28,39	16,40	23,96	25,59	24,36	28,53	17,02	25,05	NEGAT	0,45
43	NILM	30,72	16,34	19,00	28,05	26,15	24,74	13,58	21,30	NEGAT	0,29
44	NILM	30,87	16,14	21,96	24,54	28,04	21,67	15,61	23,55	NEGAT	0,17
45	NILM	26,72	17,10	22,78	29,11	24,98	27,07	13,21	23,17	NEGAT	0,35
46	NILM	28,80	16,24	21,08	21,44	26,09	19,48	14,07	21,63	NEGAT	0,16

47	NILM	27,38	16,93	22,67	30,12	26,49	26,44	15,01	22,66	NEGAT	0,25
48	NILM	28,33	16,69	20,50	22,74	26,42	23,06	14,59	20,68	NEGAT	0,11
49	NILM	25,72	18,20	23,57	22,07	25,34	15,31	14,83	21,31	NEGAT	0,35
50	NILM	25,82	16,66	23,66	25,27	27,22	23,96	16,23	23,70	NEGAT	0,16
51	NILM							37,93		NEGAT	
52	NILM	25,72	16,67	21,27	22,08	24,67	19,37	16,38	26,90	NEGAT	0,77
53	NILM	26,91	17,23	23,09	23,45	26,94	22,10	15,30	22,41	NEGAT	0,07
54	NILM	23,93	16,88	22,44	26,57	24,85	23,11	16,79	24,36	NEGAT	0,56
55	NILM	39,58	18,79	22,69	19,43	21,30	17,03	12,04	19,02	NEGAT	
56	NILM		40,64				33,20	18,59	24,29	NEGAT	
57	NILM	25,53	17,41	23,21	22,19	25,37	20,05	14,39	21,81	NEGAT	0,21
58	NILM									NEGAT	
59	NILM	28,53	16,65	20,22	19,73	25,06	20,99	14,83	23,08	NEGAT	0,49
60	NILM	28,28	16,75	20,46	21,39	24,16	27,68	15,45	23,85	NEGAT	0,47
61	NILM									NEGAT	
62	NILM	25,36	18,34	26,51	24,43	27,39	23,43	14,93	20,71	NEGAT	0,41
63	NILM	43,23	19,28	26,48	22,25	27,06	18,04	14,40	19,59	NEGAT	
64	NILM	35,75	18,30	24,95	23,22	26,27	20,64	16,04	22,82	NEGAT	

65	NILM	28,71	16,81	22,47	23,32	25,57	19,35	16,24	23,94	NEGAT	0,64
66	NILM	30,64	16,82	21,17	21,32	23,28	17,21	15,07	23,42	NEGAT	0,66
67	NILM	35,92	18,06	24,08	20,09	24,18	23,08	11,98	19,75	NEGAT	
68	NILM	32,03	18,17	25,37	23,53	28,14	25,19	13,70	20,06	NEGAT	0,47
69	NILM	31,09	17,27	22,37	21,24	24,98	18,58	16,03	22,66	NEGAT	0,30
70	NILM	27,60	16,91	24,05	23,97	28,06	25,54	18,08	24,37	NEGAT	0,19
71	NILM	33,01	17,87	22,17	19,52	22,67	16,52	13,31	21,32	NEGAT	0,42
72	NILM	24,82	16,99	21,15	20,75	22,50	14,29	14,00	22,02	NEGAT	0,73
73	NILM	24,88	16,92	21,35	20,23	23,70	16,87	13,40	20,21	NEGAT	0,38
74	NILM	23,32	16,75	20,97	20,13	21,99	18,32	13,56	21,43	NEGAT	0,57
75	NILM							32,87		NEGAT	
76	NILM		25,84	36,81	27,89	31,27	24,07	12,88	18,59	NEGAT	
77	NILM	31,41	17,90	24,22	20,41	24,72	21,32	14,24	20,81	NEGAT	0,25
78	NILM	29,16	16,98	23,32	22,45	27,45	25,13	17,05	24,62	NEGAT	0,16
79	NILM		21,81	30,24	26,62	35,24	29,04	13,47	18,48	NEGAT	
80	NILM	24,25	17,51	22,62	22,58	24,65	17,82	14,55	21,70	NEGAT	0,58
81	NILM	25,20	16,92	21,44	22,61	24,99	26,58	15,15	22,77	NEGAT	0,23
82	NILM	28,67	16,92	20,49	22,92	26,01	21,06	13,74		NEGAT	0,34

83	NILM	30,50	17,02	22,88	29,59	27,35	24,73	15,48	23,82	NEGAT	0,15
84	NILM	30,83	16,74	21,83	23,50	27,24	20,24	16,02	28,28	NEGAT	0,50
85	NILM	28,37	17,52	20,51	20,01	23,62	18,22	14,71	22,56	NEGAT	0,40
86	NILM	30,16	17,17	21,51	25,22	28,20	25,39	15,71	24,18	NEGAT	0,20
87	NILM	33,47	18,33	25,39	24,53	28,36	21,87	16,03	23,01	NEGAT	0,19
88	NILM	28,05	17,77	22,19	21,95	27,38	22,27	15,26	22,58	NEGAT	0,20
89	NILM	26,13	17,26	21,42	29,75	26,11	27,79	15,20	24,38	NEGAT	0,21
90	NILM	29,76	16,46	19,62	20,24	23,61	21,18	14,31	23,74	POSITIVE	0,51
91	NILM	29,31	17,26	20,11	23,73	24,79	21,00	14,31	23,82	NEGAT	0,67
92	NILM	29,97	17,24	21,27	23,12	25,88	26,47	15,00	23,15	NEGAT	0,14
93	NILM		20,60	29,17	24,17	30,27	24,23	13,30	18,16	NEGAT	
94	NILM	34,93	18,68	23,16	20,73	25,38	17,20	11,20	18,96	NEGAT	
95	NILM	29,81	16,60	21,73	25,03	28,21	22,99	15,08	23,51	NEGAT	0,03
96	NILM	24,39	17,18	22,00	27,20	25,97	29,19	16,94	23,85	NEGAT	0,43
97	NILM		20,11	27,17	22,18	26,90	19,25	13,05	18,38	NEGAT	
98	NILM	25,29	17,47	21,87	21,29	23,86	22,93	13,89	20,46	NEGAT	0,21
99	NILM	28,52	16,68	20,06	19,75	23,07	16,66	15,05	22,17	NEGAT	0,49
100	NILM	29,09	17,34	21,44	23,40	28,56	17,01	15,44	23,22	NEGAT	0,21

101	NILM	28,60	16,70	20,22	20,01	23,89	18,45	14,78	23,05	NEGAT	0,47
102	NILM	30,40	17,25	23,76	23,93	26,88	23,05	16,66	23,84	NEGAT	0,28
103	NILM	29,85	16,96	23,18	24,24	26,68	19,14	16,21	24,64	NEGAT	0,34
104	NILM	31,55	16,99	23,75	24,24	27,16	24,65	16,23	25,22	NEGAT	0,25
105	NILM	30,27	16,94	23,62	25,10	27,70	19,38	17,05	24,78	NEGAT	0,30
106	NILM	32,31	17,08	25,37	30,35	28,22	26,17	17,06	25,62	NEGAT	0,26
107	NILM	31,42	16,82	24,38	29,33	27,75	27,75	18,98	27,62	NEGAT	0,46
108	NILM	33,20	17,01	24,77	25,43	29,34	18,37	19,04	26,75	NEGAT	0,73
109	NILM	32,63	17,13	23,49	24,14	29,82	27,24	17,14	25,16	NEGAT	0,25
110	NILM	31,58	17,96	22,04	22,14	27,88	20,45	15,26	23,20	NEGAT	0,26
111	NILM	32,52	17,02	24,08	28,03	30,65	25,82	17,83	27,32	NEGAT	0,27
112	NILM	32,05	16,95	20,97	30,40	29,45	26,29	14,10	25,24	NEGAT	0,40
113	NILM	30,18	16,85	22,10	22,41	26,32	18,11	15,01	23,19	NEGAT	0,36
114	NILM	28,43	16,98	23,01	23,92	25,88	23,61	16,81	26,27	NEGAT	0,58
115	HSIL	23,03	16,76	27,42	29,11	23,03	31,18	17,99	23,70	NEGAT	0,65
116	HSIL	21,22	17,36	28,25	28,75	24,18	30,87	19,76	25,06	POSITIVE	0,64
117	HSIL	26,50	17,26	26,98	29,46	23,54	30,69	16,19	24,57	POSITIVE	0,71
118	HSIL	26,07	15,80	26,90	29,59	23,62	29,08	16,49	22,65	POSITIVE	0,70

119	HSIL	32,84	16,17	31,86	32,10	29,29	32,71	16,10	30,81	NEGAT	0,79
120	HSIL	26,07	16,11	25,08	26,15	24,79	28,46	12,93	18,96	NEGAT	0,85
121	HSIL	25,16	16,42	21,02	27,85	24,34	28,57	15,44	24,34	POSITIVE	0,25
122	HSIL	22,49	16,62	27,09	27,76	24,22	26,45	9,84	17,36	POSITIVE	0,98
123	HSIL	26,03	15,95	26,13	28,06	23,85	24,56	13,17	20,05	POSITIVE	1,00
124	HSIL	24,17	16,24	27,28	28,15	22,95	25,77	14,07	20,36	POSITIVE	1,00
125	HSIL	28,57	16,09	26,24	26,69	25,74	29,01	14,07	21,31	POSITIVE	0,85
126	HSIL	31,68	16,25	26,35	29,47	28,13	24,35	15,25	22,26	POSITIVE	0,52
127	HSIL	23,23	16,13	26,74	28,39	23,62	25,88	12,24	18,72	POSITIVE	0,99
128	HSIL	24,96	15,64	27,02	27,90	24,91	21,24	13,18	18,79	POSITIVE	0,99
129	HSIL	25,20	15,77	26,66	28,27	23,08	23,60	13,80	19,95	POSITIVE	0,99
130	HSIL	24,46	15,43	26,06	27,06	24,99	22,28	13,46	19,50	POSITIVE	0,89
131	HSIL	30,35	16,12	19,41	20,35	26,37	20,95	14,41	23,04	NEGAT	0,40
132	HSIL	21,25	18,01	27,13	28,32	23,82	27,45	8,82	17,40	POSITIVE	0,97
133	HSIL	26,40	16,79	25,83	27,19	25,42	23,12	12,04	19,03	POSITIVE	0,91
134	HSIL	29,33	16,59	20,65	21,19	24,09	19,79	13,86	23,28	POSITIVE	0,40
135	HSIL	23,46	16,67	27,27	29,21	22,82	25,01	13,94	20,73	POSITIVE	1,00
136	HSIL	29,22	16,49	19,38	20,08	23,21	14,40	13,66	24,17	POSITIVE	0,51

137	HSIL	33,48	16,90	23,45	25,40	29,22	18,05	18,01	27,70	NEGAT	0,55
138	HSIL	32,56	16,60	20,07	19,19	24,05	14,71	14,47	23,55	NEGAT	0,48
139	HSIL	22,69	16,46	27,68	28,08	25,38	26,41	13,48	19,97	POSITIVE	1,00
140	HSIL	24,37	16,33	27,07	27,28	22,46	24,25	12,37	19,19	POSITIVE	0,99
141	HSIL	25,81	16,53	26,11	28,35	23,66	25,85	13,59	20,57	POSITIVE	0,97
142	HSIL	25,33	16,27	26,21	27,33	23,30	27,03	12,55	19,64	POSITIVE	0,99
143	HSIL	25,61	16,21	27,06	28,25	22,43	23,84	13,20	19,71	POSITIVE	0,99
144	HSIL	23,29	16,80	28,27	28,71	23,89	26,27	12,26	18,24	POSITIVE	0,99
145	HSIL	24,81	16,82	26,23	28,50	24,89	27,04	10,35	16,68	POSITIVE	0,97
146	HSIL	28,81	16,16	28,19	29,95	26,42	26,70	14,61	20,76	POSITIVE	0,99
147	HSIL	20,53	17,75	26,97	28,10	23,21	28,10	11,44	18,21	NEGAT	0,95
148	HSIL	22,94	16,49	27,52	28,80	23,25	25,73	12,48		POSITIVE	0,82
149	HSIL	26,73	15,96	27,88	29,24	23,24	27,46	14,75	21,03	POSITIVE	0,98
150	HSIL	28,71	16,34	26,79	30,12	25,51	24,68	13,98	20,28	POSITIVE	1,00
151	HSIL	28,02	16,40	22,03	23,89	24,92	20,58	14,98	23,61	POSITIVE	0,62
152	HSIL	25,60	16,89	27,37	28,78	24,56	27,67	12,29	18,31	POSITIVE	0,98
153	HSIL	27,15	16,76	26,76	29,21	24,41	27,33	14,80	21,26	POSITIVE	0,99
154	HSIL	28,74	16,73	28,24	28,39	26,01	26,80	14,77	21,15	POSITIVE	0,98

155	HSIL	31,25	17,07	20,82	31,09	29,31	24,53	15,05	25,84	POSITIVE	0,24
156	HSIL	29,61	17,97	22,57	19,67	24,13	13,44	14,09	20,87	NEGAT	0,38
157	HSIL	25,87	16,38	19,25	18,59	21,18	13,71	12,18	19,12	NEGAT	0,31
158	HSIL	25,62	16,15	20,09	21,10	22,72	16,12	12,61	20,08	POSITIVE	0,61
159	HSIL	26,37	15,88	27,21	28,90	24,00	25,44	15,20	20,32	POSITIVE	1,00
160	HSIL	25,49	16,34	21,10	21,77	23,51	15,55	13,03	19,44	POSITIVE	0,37
161	HSIL		20,21	27,08	22,85	24,54	14,12	13,59	19,14	NEGAT	
162	HSIL	31,42	16,68	20,44	22,29	25,71	18,52	15,56	22,98	NEGAT	0,72
163	HSIL		19,95	26,89	22,10	26,41	15,06	13,57	20,20	NEGAT	
164	HSIL	24,06	16,36	22,89	22,31	25,09	15,01	15,25	26,07	POSITIVE	0,49
165	HSIL	25,00	16,60	22,05	27,98	28,29	31,37	15,94	28,25	POSITIVE	0,23
166	HSIL	23,84	16,68	20,68	21,05	23,41	16,49	13,07	20,60	NEGAT	0,27
167	HSIL	24,56	16,43	19,65	19,61	23,02	16,41	12,99	21,42	NEGAT	0,26
168	HSIL	24,64	17,84	21,54	26,08	27,00	26,55	14,08	21,15	NEGAT	0,21
169	HSIL	28,84	17,81	19,82	19,17	23,75	15,95	12,68	20,00	NEGAT	0,18
170	HSIL	26,99	16,56	19,47	21,89	24,68	19,57	12,74	20,50	NEGAT	0,05
171	HSIL	24,10	16,50	20,82	21,34	25,47	19,63	13,89	23,02	POSITIVE	0,25
172	HSIL	37,84	18,30	29,17	27,19	27,88	16,38	18,08	23,72	POSITIVE	

173	HSIL	27,27	16,91	19,88	20,69	22,83	18,00	13,07	21,51	NEGAT	0,51
174	HSIL	37,75	18,30	24,92	22,17	24,63	16,86	14,46	22,82	NEGAT	
175	HSIL	27,00	17,19	22,83	24,15	25,05	24,18	11,17	18,06	POSITIVE	0,61
176	HSIL	29,97	16,96	28,09	29,77	26,31	28,06	15,16	21,92	NEGAT	1,00
177	HSIL	34,02	18,12	25,04	25,02	28,55	20,47	16,59	24,32	NEGAT	
178	HSIL	26,83	17,11	27,20	29,09	24,93	25,58	12,50	19,14	NEGAT	1,00
179	HSIL	23,92	18,20	26,23	27,18	26,29	25,56	15,27	22,87	NEGAT	0,46
180	HSIL	28,93	18,15	25,68	29,59	26,73	23,04	12,33	19,68	POSITIVE	0,82
181	HSIL	28,03	16,58	24,63	28,02	27,09	24,66	12,70	19,37	POSITIVE	0,84
182	HSIL	22,47	16,91	28,56	29,18	22,64	22,54	16,06	22,62	POSITIVE	0,91
183	HSIL	25,53	16,42	33,54	29,84	23,06	29,38	16,32	23,01	POSITIVE	0,60
184	HSIL	26,49	16,41	22,45	23,81	27,33	18,57	16,76	31,60	POSITIVE	0,49
185	HSIL	34,71	17,79	22,41	18,64	22,74	13,22	12,04	19,69	NEGAT	
186	HSIL	24,84	16,69	27,70	27,96	24,35	28,64	13,07	19,83	POSITIVE	0,97
187	HSIL	28,17	16,75	19,80	25,68	26,61	23,16	14,45	23,40	NEGAT	0,33
188	HSIL	25,39	16,43	20,81	22,43	24,92	20,45	15,12	23,54	POSITIVE	0,60
189	HSIL		18,84	24,90	21,90	24,32	15,92	13,63	20,00	NEGAT	
190	HSIL	26,79	16,29	25,47	27,15	24,14	21,67	13,80	20,24	POSITIVE	0,85

191	HSIL	22,67	16,55	26,56	27,59	23,57	18,65	12,87	18,54	POSITIVE	0,98
192	HSIL	26,08	16,32	27,07	27,93	26,17	27,17	15,06	21,63	POSITIVE	0,85
193	HSIL	32,95	17,29	22,61		25,46	18,25	17,92	25,09	NEGAT	0,70
194	HSIL	35,00	17,10	25,56	26,65	28,41	25,69	20,27	27,98	NEGAT	
195	HSIL	34,30	16,49	24,33	25,31	28,13	22,65	19,23	25,96	POSITIVE	
196	HSIL	27,70	16,78	24,40	25,49	24,92	24,99	18,24	25,91	POSITIVE	0,47
197	HSIL	33,22	16,72	24,81	25,98	29,05	25,49	18,81	26,43	POSITIVE	0,29
198	HSIL	33,32	16,63	23,16	25,34	27,69	23,57	18,35	26,30	NEGAT	0,88
199	HSIL	34,03	16,91	23,66	26,15	26,39	19,39	20,51	28,43	POSITIVE	
200	HSIL	32,05	16,79	21,75	24,56	24,47	16,95	16,75	24,78	NEGAT	0,92
201	HSIL	33,62	16,97	27,48	29,10	30,47	25,87	21,34	27,63	POSITIVE	0,62
202	HSIL	30,25	16,70	22,22	23,78	25,37	17,46	17,94	25,79	POSITIVE	0,92
203	HSIL	33,81	16,80	26,84	28,09	28,85	25,16	21,05	27,69	POSITIVE	0,77
204	HSIL	35,27	17,05	27,45	28,78	29,58	26,86	21,38	28,04	POSITIVE	
205	HSIL	33,12	15,79	22,90	25,13	25,56	16,69	18,10	24,77	POSITIVE	0,89
206	HSIL	32,05	16,22	21,19	23,91	23,93	17,34	16,40	23,56	POSITIVE	0,93
207	HSIL	28,32	16,03	21,08	22,41	22,21	19,54	15,22	22,15	NEGAT	0,69
208	HSIL	30,41	16,18	23,25	25,65	23,87	16,49	17,63	24,10	POSITIVE	0,69

209	HSIL	32,96	16,21	22,40	24,22	26,30	22,50	17,01	24,74	POSITIVE	0,68
210	HSIL	33,17	16,04	23,06	24,15	28,34	22,11	18,24	24,80	NEGAT	0,61
211	HSIL	32,61	15,88	21,17	22,49	23,83	13,67	16,33	23,73	NEGAT	0,95
212	HSIL	33,04	15,94	21,75	22,88	26,52	25,07	16,23	23,76	NEGAT	0,20
213	HSIL	33,51	15,95	22,11	25,80	28,81	26,11	18,02	24,60	POSITIVE	0,50
214	HSIL	33,12	16,00	22,54	23,87	26,22	21,05	17,19	24,25	POSITIVE	0,58
215	HSIL	32,20	15,98	21,27	24,41	24,62	16,30	17,00	24,77	NEGAT	0,93
216	HSIL	30,86	16,00	21,69	23,88	25,97	23,43	16,37	24,36	POSITIVE	0,50
217	HSIL	32,12	16,74	21,97	24,99	24,59	16,76	17,76	25,21	POSITIVE	0,99
218	HSIL	31,73	16,89	24,66	25,88	26,73	24,20	18,37	25,93	POSITIVE	0,58
219	HSIL	32,94	16,75	22,65	23,82	24,72	18,24	18,00	26,05	POSITIVE	0,92
220	HSIL	31,26	16,60	21,71	23,66	24,92	18,76	15,55	23,16	POSITIVE	0,58
221	HSIL	33,18	16,68	21,59	24,37	28,42	22,57	18,06	26,00	NEGAT	0,79
222	HSIL	32,70	16,69	20,30	22,00	25,61	18,18	16,03	24,12	POSITIVE	0,75
223	HSIL	31,57	16,87	21,71	25,64	26,08	20,01	17,23	24,81	POSITIVE	0,76
224	HSIL	33,13	16,72	22,72	23,88	26,61	20,53	18,42	26,18	POSITIVE	0,64
225	HSIL	32,91	17,18	19,83	22,47	24,85	18,11	15,55	23,86	POSITIVE	0,87
226	HSIL	25,06	17,39	27,92	28,63	25,14	24,51	10,03	17,64	POSITIVE	0,99
